# Temporal trends in primary care-recorded psychiatric diagnoses and psychotropic medication prescribing among children and young people in the UK: a population-based study

**DOI:** 10.3399/BJGP.2024.0804

**Published:** 2025-08-12

**Authors:** Alex M Trafford, Matthew J Carr, Darren M Ashcroft, Carolyn A Chew-Graham, Emma Cockcroft, Lukasz Cybulski, Emma Garavini, Shruti Garg, Louise Hussey, Thomas Kabir, Nav Kapur, Rachel K Temple, Roger T Webb, Pearl LH Mok

**Affiliations:** 1 Centre for Pharmacoepidemiology & Drug Safety, Division of Pharmacy & Optometry, Manchester Academic Health Sciences Centre, University of Manchester, Manchester, UK; 2 NIHR Greater Manchester Patient Safety Research Collaboration, University of Manchester, Manchester, UK; 3 Faculty of Medicine & Health Sciences, Keele University, Keele, UK; 4 Department of Health & Community Sciences, University of Exeter, Exeter, UK; 5 Division of Insurance Medicine, Karolinska Institutet, Stockholm, Sweden; 6 McPin Foundation, London, UK; 7 Division of Psychology & Mental Health, Manchester Academic Health Sciences Centre, University of Manchester, Manchester, UK; 8 Royal Manchester Children’s Hospital, Central Manchester University Hospitals NHS Foundation, Manchester, UK; 9 Departments of Experimental Psychology & Psychiatry, University of Oxford, Oxford, UK; 10 Mersey Care NHS Foundation Trust, Liverpool, UK

**Keywords:** ADHD, COVID-19, mental health, patient involvement, primary health care

## Abstract

**Background:**

Despite growing concerns about young people’s mental health, it remains unclear how rates of psychiatric diagnoses and psychotropic medication prescribing have changed.

**Aim:**

To investigate temporal trends in UK primary care-recorded incidence of (1) psychiatric diagnoses: attention deficit hyperactivity disorders (ADHD), autism spectrum conditions, anxiety disorders, depression, substance misuse, and personality disorders, and (2) psychotropic medication prescribing, in individuals aged 1–24 years.

**Design and setting:**

This was a population-based study using primary care data from the Clinical Practice Research Datalink.

**Method:**

The monthly incidence of each outcome from January 2010 to March 2022 were calculated. Negative binomial regression was used to predict expected incidence rates when the COVID-19 pandemic began in March 2020, based on antecedent trends. Observed and predicted (that is, expected) rates were compared.

**Results:**

In the 2 years following March 2020, the incidence of ADHD diagnoses in females was 24.7% (95% confidence interval = 11.9 to 38.9%) greater than the expected incidence rate predicted from the trends before the pandemic. The increase in ADHD diagnoses occurred more commonly for females aged 20–24 years, followed by those aged 17–19 years, as well as among females from less deprived areas. Similar trends were observed for ADHD medications. Observed rates of other outcomes, including common mental illnesses, were below or close to the expected levels, with the differentials between observed and expected rates being greater for males than females.

**Conclusion:**

Increased ADHD awareness may partly explain the study’s findings. However, the fall in other diagnoses may reflect barriers to accessing health services at the height of the pandemic. Early identification and timely treatment of mental health difficulties and neurodevelopmental conditions are crucial.

## How this fits in

Many studies have reported that there has been an adverse impact on children and young people’s mental health from the COVID-19 pandemic, but there is a lack of evidence about whether this has led to an increase in psychiatric diagnoses and psychotropic medication prescribing. This study of UK primary care data on individuals aged 1–24 years found that there was a substantial increase in the incidence rates of attention deficit hyperactivity disorder (ADHD) diagnosis and ADHD medication in the first 2 years of the pandemic among young adult females, especially those in less deprived areas. However, no increase was found for other psychiatric diagnoses or psychotropic medication prescribing investigated. Timely diagnosis and access to treatments are needed to prevent exacerbations of mental health difficulties and neurodevelopmental conditions.

## Introduction

Mental health difficulties commonly have onset during childhood or adolescence, with between half and three-quarters of lifetime cases of individuals with these conditions being diagnosed by people’s early 20s.^1–3^ Children born in the UK between 2000 and 2002 were found to not only have higher levels of emotional problems than those born a decade previously, but also earlier emergence of problems.^4^ A study of UK primary care records reported a substantial rise in the number of psychiatric diagnoses and self-harm episodes among children and adolescents between 2003 and 2018, with the largest increase in incidence rates being observed for attention deficit hyperactivity disorders (ADHD) and autism spectrum conditions among girls.^5^ The number of prescriptions for antidepressants, ADHD medications, and antipsychotics issued by GPs for children and adolescents also rose during this period.^6–8^

Many challenges that young people face might have been exacerbated during the COVID-19 pandemic. The Mental Health of Children and Young People in England survey found that one in nine children aged 7 to 16 years had a probable mental disorder in 2017. This rose to one in six in 2020, with the rates remaining stable through the pandemic.^9^ However, although there has been a large body of literature reporting a rise in negative psychological symptoms and mental health difficulties among some children and young people during this time, it remains unclear how rates of psychiatric diagnoses in the UK have changed.^10–14^ In a study using UK primary care data, the authors of the current study reported a substantial increase in the incidence of eating disorder diagnoses and self-harm among teenage girls since the pandemic began.^15^ The current study investigated the temporal trends in UK primary care-recorded incidence of: ADHD, autism spectrum conditions, anxiety disorders, depression, substance misuse, personality disorders, and psychotropic medication prescriptions in children and young people aged 1–24 years. To account for repeat medications, this study also investigated event rates for psychotropic medication prescribing. To quantify any changes observed since the pandemic started, observed rates from March 2020 were compared with the trends predicted using pre-pandemic data.

## Method

### Study design and population

A population-based study was conducted using data from the Clinical Practice Research Datalink (CPRD), a database of anonymised electronic health records from UK primary care (Supplementary Information S1). Data from the Aurum dataset were used to analyse temporal trends in England and the GOLD dataset for investigations in Northern Ireland, Scotland, and Wales.^16,17^ Linked practice-level Index of Multiple Deprivation quintile data — a composite measure of socioeconomic deprivation based on the postcode of the general practice — were used.^18^

Patient records between 1 January 2010 and 31 March 2022 were investigated. Pre-pandemic data before March 2020 were included to investigate long-term trends (see ‘Statistical analyses’ subsection), but the focus of the reporting is January 2019 to March 2022. Contribution to time at risk and outcome counts began following the last to occur of: 1 year of continuous registration in a practice, 1 year of contribution to a practice deemed to be up to CPRD standard, or first birthday. Follow-up of eligible individuals ceased at the first to occur of: date of outcome of interest, 25th birthday, end of practice registration, last practice data-collection date, or death. For prescribing event rates, the date of outcome was not considered as a follow-up endpoint.

### Outcomes

Primary outcomes were monthly incidence rates of: ADHD (1–24 years of age), autism spectrum conditions (1–24 years of age), anxiety disorders (6–24 years of age), depression (6–24 years of age), substance misuse (17–24 years of age), personality disorders (17–24 years of age), and psychotropic medications prescribing (6–24 years of age). Prescribing was investigated as a composite outcome, with ADHD medications, anxiolytics, antidepressants, antipsychotics, and benzodiazepines additionally examined as individual outcomes. Diagnostic records were identified from SNOMED/EMIS/Read codes. Medications were identified by product codes.

Secondary outcomes included monthly event rates for prescribing. Unlike incidence rates where only the first prescription of the outcome of interest was considered, event counts included the first and any subsequent prescriptions. In addition, because some of the Read codes descriptions can seem ambiguous, the current study also compared incidence of depression identified by ‘stringent’ versus ‘inclusive’ codes. ‘Stringent’ codes are those that are perceived to indicate a high likelihood of correctly ascertaining a depression diagnosis, such as, ‘depression’, ‘postnatal depression’, and ‘depressive episode’. ‘Inclusive’ codes include all ‘stringent’ codes, as well as those that are more ambiguous such as ‘low/sad mood’ and ‘feeling unhappy’.^5^ All code lists were compiled using the CPRD code browser tool. The search terms were developed with, and the resulting code lists reviewed and refined by, three of the authors (the third author who is a pharmacist, the fourth author who is an academic GP with expertise in mental health, and the eighth author who is a child and adolescent psychiatrist). Code lists are available in Supplementary Information S2–S11.

### Statistical analyses

Incidence and event rates were calculated monthly from January 2010 through March 2022, with the denominator being the aggregated person-months at risk, and the numerator being the number of outcome events. Rates were calculated by sex, age group, and deprivation quintiles. To quantify changes in outcome occurrence after the start of the pandemic, observed monthly incidence and event rates from March 2020 onwards were compared with expected monthly rates. Expected rates following the pandemic’s onset were predicted using negative binomial regression based on monthly rates observed between January 2010 and February 2020. Direct comparison between observed and expected rates were expressed as the percentage difference between the two. See Supplementary Information S12 for further information. Analyses were performed using Stata v16.

### Patient and public involvement and engagement

The authors worked with the McPin Foundation, who convened an advisory group of young people aged 13–25 years with lived experience of mental health difficulties, and with parents and carers of young people.^19^ The authors met and consulted with these patient and public involvement and engagement (PPIE) advisers regularly throughout the study. The study’s PPIE partners particularly helped the authors to interpret the findings and they advised on key messages, recommendations, and dissemination. For detailed information on how the authors collaborated with the PPIE members in this study, see Cockcroft *et al*.^19^ A stakeholder group of professionals working with young people also informed the study.

## Results

### Study cohort

A total of 12 371 319 individuals aged 1–24 years were included in the study cohort between 1 January 2010 and 31 March 2022. This consisted of10 362 294 individuals from 1475 practices in England and 2 009 025 individuals from 406 practices in Scotland, Wales, and Northern Ireland. Supplementary Figures S1 and S2 show how the two cohorts were delineated, respectively. Table 1 shows their demographic characteristics as of 1 March 2020.

**Table 1.** Demographic characteristics of the study population as of 1 March 2020.^a^

	Practices in England(*N* = 1414*)n(%)*	Practices in Northern Ireland, Scotland, and Wales(*N* = 377)*n(%)*	Practices in UK(*N* = 1791)*n(%)*
**All patients**	3 931 279	754 834	4 686 113
Sex			
Female	1 943 067 (49.4)	370 544 (49.1)	2 313 611 (49.4)
Male	1 988 212 (50.6)	384 290 (50.9)	2 372 502 (50.6)
**Age group, years**			
1–5	761 664 (19.4)	145 678 (19.3)	907 342 (19.4)
6–9	648 577 (16.5)	128 469 (17.0)	777 046 (16.6)
10–12	485 922 (12.4)	100 050 (13.3)	585 972 (12.5)
13–16	601 778 (15.3)	124 039 (16.4)	725 817 (15.5)
17–19	452 436 (11.5)	86 467 (11.5)	538 903 (11.5)
20–24	980 902 (25.0)	170 131 (22.5)	1 151 033 (24.6)
**IMD quintile**			
1 (least deprived)	625 694 (15.9)	124 106 (16.4)	749 800 (16.0)
2	642 009 (16.3)	131 989 (17.5)	773 998 (16.5)
3	781 991 (19.9)	152 388 (20.2)	934 379 (19.9)
4	922 911 (23.5)	181 172 (24.0)	1 104 083 (23.6)
5 (most deprived)	958 674 (24.4)	165 179 (21.9)	1 123 853 (24.0)

^a^The numbers of practices reported in this table were the numbers contributing data to CPRD as of 1 March 2020, whereas the numbers reported in the text (*n* =1475 in England; 406 in Scotland, Wales, and Northern Ireland) were the numbers pertaining to the period 1 January 2010 to 31 March 2022.

CPRD = Clinical Practice Research Datalink; IMD = Index of Multiple Deprivation;

### Psychiatric diagnoses

**Figure 1.**
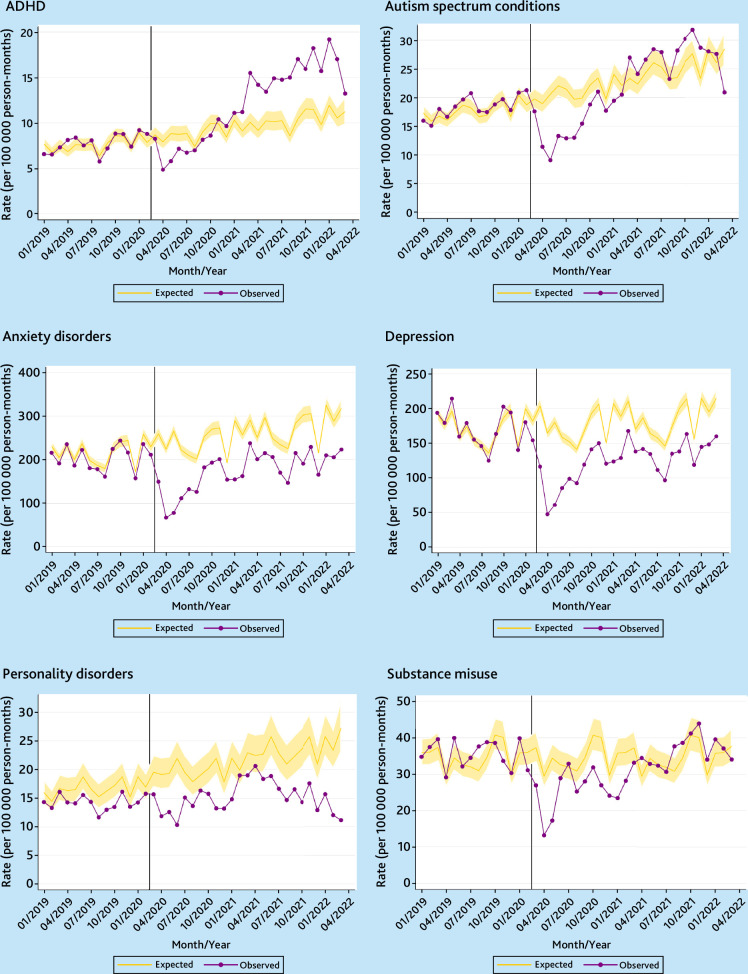
Temporal trends in monthly observed versus expected incidence rates of psychiatric diagnoses in the UK for females (January 2019 to March 2022). Vertical line denotes 1 March 2020. ADHD = attention deficit hyperactivity disorder.

**Figure 2.**
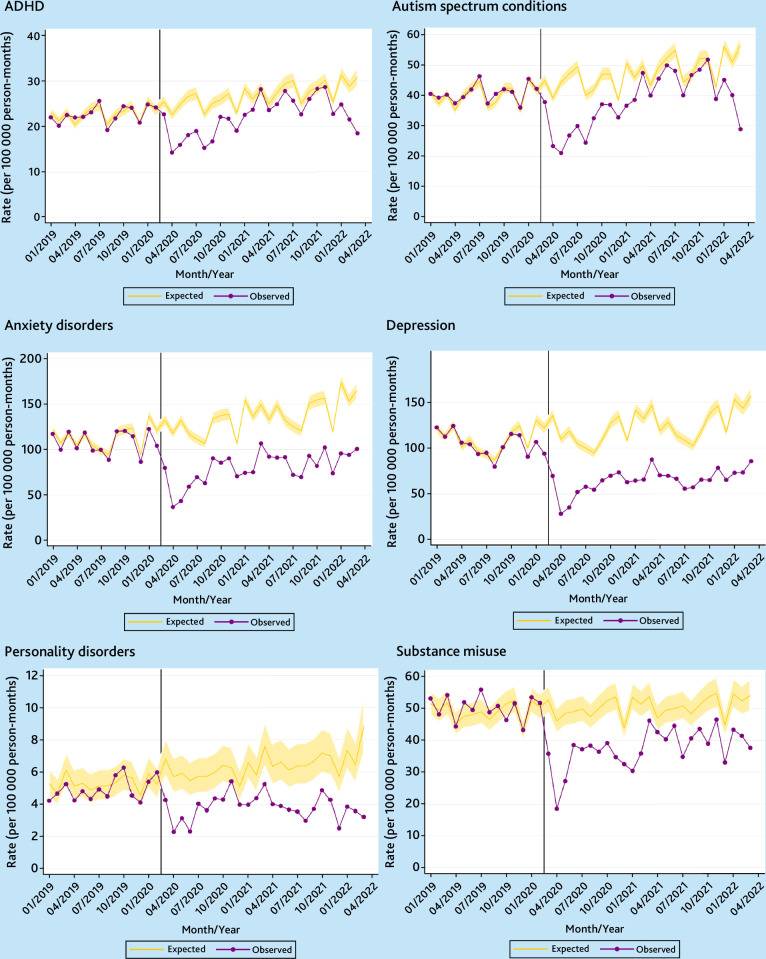
Temporal trends in monthly observed versus expected incidence rates of psychiatric diagnoses in the UK for males (January 2019 to March 2022). ADHD = attention deficit hyperactivity disorder. Vertical line denotes 1 March 2020.

The monthly observed and expected incidence rates of psychiatric diagnoses for females and males between January 2019 and March 2022 are presented in Figures 1 and 2, respectively. Temporal trends for the whole study period January 2010 through March 2022 are shown in Supplementary Figures S3 and S4 for females and males, respectively. Across this observation period, incidence of ADHD, autism spectrum conditions, and substance misuse were consistently higher for males than for females, whereas anxiety disorders, depression, and personality disorders were more commonly diagnosed in females.

Coinciding with the implementation of initial COVID-19 restrictions, incidence rates across all diagnostic outcomes fell sharply in April 2020 for both sexes. Following this initial reduction, incidence rates of ADHD in females rose substantially, exceeding expected levels by February 2021. For the 2 years since the pandemic began, diagnoses of ADHD in females were 24.7% (95% confidence interval (CI) = 11.9 to 38.9%) greater than expected (Table 2). The increases were attributable to females aged 20–24 years and, to a lesser extent, those aged 17–19 years, with the incidence being 158.6% (95% CI = 83.8 to 264.3%) and 52.8% (95% CI = 4.8 to 122.4%), respectively, greater than expected (Supplementary Table S1). In the year before the pandemic, ADHD was more commonly diagnosed among females in the least deprived (quintile 1) than in the most deprived (quintile 5) areas, and this differential has widened since (Supplementary Figure S5 and Table S2).

**Table 2.** Difference between expected versus observed number of psychiatric diagnoses and prescription of psychotropic medications by sex in the UK (March 2020 to March 2022)

	Expected number of psychiatric diagnoses/prescriptions (95% CI)	Observed number of psychiatric diagnoses/ prescriptions	Percentage difference in observed versus expected number of psychiatric diagnoses/prescriptions, % (95% CI)
**Females**			
ADHD	4715 (4233 to 5251)	5880	24.7 (11.9 to 38.9)
Autism spectrum conditions	11 191 (10 246 to 12 223)	10 468	−6.4 (−14.3 to 2.1)
Anxiety disorders	92 174 (86 359 to 98 380)	61 226	−33.5 (−37.7 to −29.1)
Depression	65 139 (61 423 to 69 081)	44 388	−31.8 (−35.7 to −27.7)
Personality disorders	3481 (3010 to 4025)	2451	−29.5 (−39.1 to −18.5)
Substance misuse	5381 (4796 to 6037)	4828	−10.2 (−20.0 to 0.6)
All psychotropic medications	75 324 (72 104 to 78 688)	69 509	−7.7 (−11.6 to −3.5)
ADHD medications	3106 (2749 to 3509)	3544	14.1 (0.9 to 28.9)
Anxiolytics	21 233 (20 195 to 22 324)	18 505	−12.8 (−17.1 to −8.3)
Antidepressants	67 586 (64 267 to 71 077)	60 285	−10.8 (−15.1 to −6.1)
Antipsychotics	17 924 (16 941 to 18 964)	16 931	−5.5 (−10.7 to 0.0)
Benzodiazepine	10 421 (9836 to 11 041)	7668	−26.4 (−30.5 to −22.0)
**Males**			
ADHD	13 469 (12 691 to 14 294)	11 155	−17.1 (−21.9 to −12.1)
Autism spectrum conditions	23 310 (22 124 to 24 560)	18 736	−19.6 (−23.7 to −15.3)
Anxiety disorders	53 264 (50 390 to 56 303)	31 257	−41.3 (−44.4 to −37.9)
Depression	48 931 (46 030 to 52 015)	25 311	−48.2 (−51.3 to −45.0)
Personality disorders	1081 (894 to 1307)	650	−39.8 (−50.2 to −27.2)
Substance misuse	8208 (7534 to 8943)	6104	−25.6 (−31.7 to −18.9)
All psychotropic medications	58 575 (56 162 to 61 093)	45 169	−22.8 (−26.0 to −19.5)
ADHD medications	9612 (8932 to 10 343)	7799	−18.8 (−24.5 to −12.6)
Anxiolytics	19 273 (18 297 to 20 302)	15 988	−17.0 (−21.2 to −12.6)
Antidepressants	46 494 (44 242 to 48 861)	33 506	−27.9 (−31.4 to −24.2)
Antipsychotics	9199 (8595 to 9845)	7290	−20.7 (−25.9 to −15.1)
Benzodiazepine	6548 (6144 to 6979)	4868	−25.6 (−30.2 to −20.7)

ADHD = attention deficit hyperactivity disorder. CI = confidence interval.

Although ADHD incidence rate in males returned to the expected level a year after the initial lockdown (Figure 2), rates were 17.1% (95% CI = 12.1 to 21.9%) lower than expected across the period as a whole (Table 2). Similarly, diagnoses of autism spectrum conditions in females rose following the initial reduction in April 2020, surpassing the expected rates in March 2021, but there was no significant difference between the observed and expected rates in the 2 years following the pandemic’s onset.

This pattern of incidence rates remaining below or close to the expected levels during the pandemic’s first 2 years was found for all other diagnostic groups, for both sexes and across all age groups and deprivation quintiles (Figures 1 and 2,Table 2, and Supplementary Tables S1–S4). The reduction in incidence was more pronounced for males than females. Between March 2020 and March 2022, incidence of depression was 48.2% (95% CI = 45.0 to 51.3%) lower than expected for males and 31.8% (95% CI = 27.7 to 35.7%) lower for females. Supplementary Figure S6 shows that, for both sexes, the drop in depression diagnoses was largely attributed to the fall in the use of ‘inclusive’ coding. Diagnoses in females based on ‘stringent’ codes actually remained close to the expected levels since October 2020. However, the use of ‘stringent’ codes remained consistently below the expected values for males.

### Psychotropic medication prescriptions

**Figure 3.**
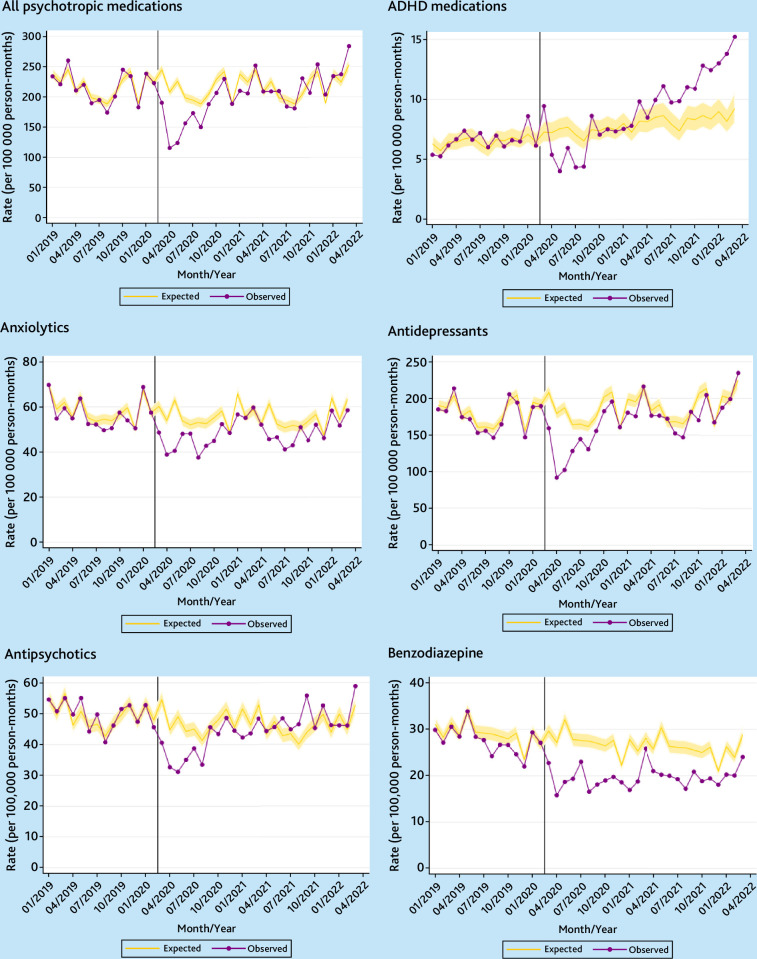
Temporal trends in monthly observed versus expected incidence rates of prescription for psychotropic medications in the UK for females (January 2019 to March 2022). Vertical line denotes 1 March 2020.

**Figure 4.**
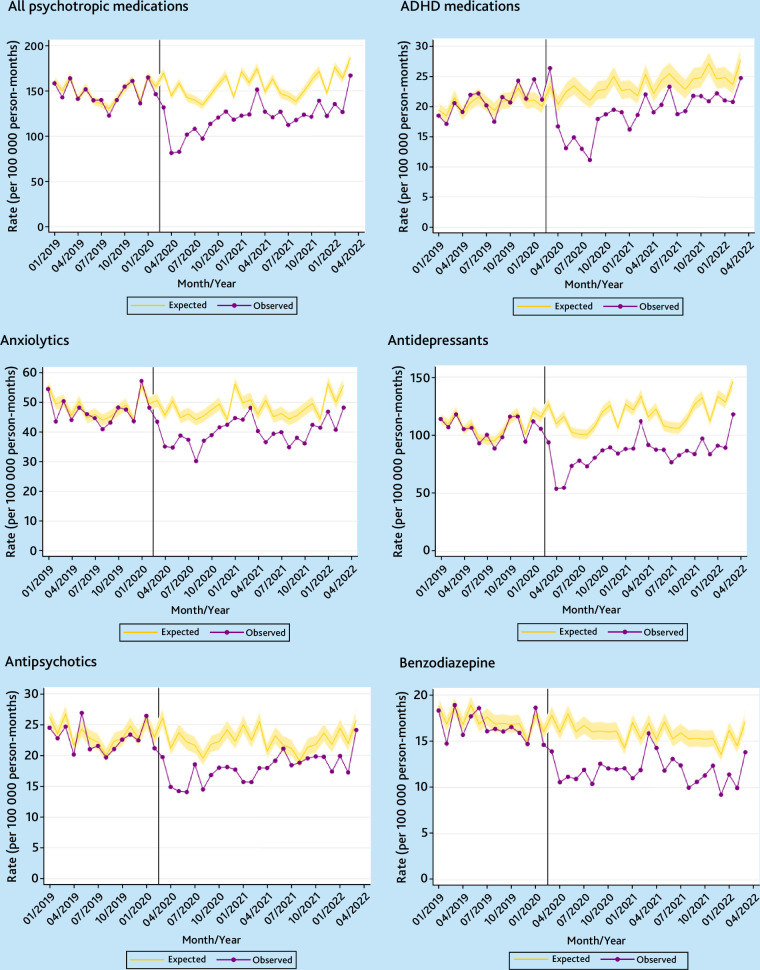
Temporal trends in monthly observed versus expected incidence rates of prescription for psychotropic medications in the UK for males (January 2019 to March 2022). Vertical line denotes 1 March 2020.

As with psychiatric diagnoses, there was a sharp fall in the incidence of psychotropic medication prescribing in April 2020 (Figures 3 and 4). In the following 2 years, incidence was 7.7% (95% CI = 3.5 to 11.6%) lower than expected for females and 22.8% (95% CI = 19.5 to 26.0%) lower for males (Table 2). The reductions in overall incident prescribing were found across almost all age groups and for all deprivation quintiles (Supplementary Tables S5–S8).

Psychotropic medications, except those for ADHD, were more commonly prescribed for females than for males. As with the substantial rise in incidence rates of ADHD observed among females, the incidence of ADHD medication prescribing rose for females and were 14.1% (95% CI = 0.9 to 28.9) greater than expected in the 2 years since March 2020. The increase was driven by those aged 20–24 years, and those from practices in less deprived areas (Supplementary Tables S5 and S6).

Incidence rates for all other types of psychotropic medications prescribing were below the expected levels, with the drop being greater for males than for females, except for benzodiazepines for which the difference between observed and expected rates were similar for both sexes (Table 2).

Supplementary Figures S7 and S8 show the sex-specific event rates of psychotropic medications prescribing. The rates since March 2020 were close to the expected levels for females, but significantly lower than expected for males, although the difference was smaller than for incident prescribing (Table S9 and Table 2). Unlike for diagnostic and incidence prescribing rates, there was no significant increase in the total number of ADHD medications issued for females.

## Discussion

### Summary

The study found a sharp rise in ADHD diagnoses among females aged 17–24 years in the 2 years following the start of the pandemic, especially among those from less deprived areas. Similar trends were observed for incident ADHD medication prescribing. However, rates of other diagnostic and prescribing outcomes, including depression and anxiety disorders, were either below or close to the expected levels, with the differentials between observed and expected rates being greater for males than females. For both sexes, the reduction in depression diagnoses was attributed more to the fall in the use of ‘inclusive’ than of ‘stringent’ codes.

### Strengths and limitations

To the authors’ knowledge, this is the largest study of temporal trends of primary care-recorded psychiatric diagnoses and psychotropic medication prescribing for children and young people in the UK before and during the first 2 years of the COVID-19 pandemic. Other similar UK time series studies have focused on depression and anxiety disorders, prescriptions for antidepressants and anxiolytics, or have more limited geographical coverage.^20–22^ For depression, this study additionally investigated the use of ‘stringent’ versus ‘inclusive’ diagnostic coding. The current study was conducted with strong patient and public involvement.^19^

The CPRD is a large longitudinal primary care database with records from a fifth of general practices in the UK.^16,17^ It is broadly representative of the UK’s population and includes all prescriptions issued in the participating practices. However, it only captures diagnoses recorded in primary care as well as those made in secondary care and private specialist clinics that were reported to the patients’ GPs. Any cases not reported or people not presenting to health services are not coded. The observed number of outcome cases that have been reported in the current study would therefore have been affected by factors such as health-seeking behaviour by individuals, access to care, general practice referral thresholds, and diagnostic and recording practices in both primary and secondary care. As such, the incidence of recorded diagnoses reported may differ from the temporal trends in mental health problems experienced in the population. In addition, the authors had no information about the indications for the prescriptions nor information on prescriptions issued in secondary care. It also was not possible to reliably stratify the analyses by ethnic group because of a large amount of missing data in the primary care study cohort.

### Comparison with existing literature

Foley *et al* reported that GP contacts with children and young people dropped by 41% during the first COVID-19 ‘lockdown’ in March 2020 compared with previous years.^23^ The sharp fall in incidence rates that was observed in the current study across all outcomes in the pandemic’s early months was consistent with this and other studies, suggesting a change in health-seeking behaviour during this time.^24–26^ Despite there being much reporting of the negative impact of the pandemic on children and young people’s mental health,^10–14^ with the exception of ADHD for females, the recorded incidence of all other outcomes investigated here remained below the expected levels during the first 2 years of the pandemic. The same author group has previously reported a substantial increase in incidence of self-harm episodes and eating disorder diagnoses among teenage girls during the same period.^15^ However, in the current study, no increase in diagnostic rates for common mental illnesses were found.

As in this study, an increase in people with ADHD in recent years has also been observed in the US.^27^ ADHD is a highly heritable childhood-onset neurodevelopmental condition.^28^ Research has shown that many children meeting diagnostic criteria for ADHD never received a clinical diagnosis, with one key determinant being whether the symptoms were noticed by the child’s family or school.^29^ Mitigation measures imposed during the pandemic might have exacerbated ADHD symptoms making them more noticeable,^27^ but this is unlikely to explain the increase in diagnoses being found in young adult females only. Another, probably more likely, explanation is the rising awareness of ADHD especially via social media such as TikTok, with ADHD being one of the most popular health topics to view.^30^ Research on digital media use and discussions with the study’s advisory groups indicated that girls were more likely than boys to use social media, whereas boys spent more time gaming.^31^ ADHD was also the second most viewed health condition on the NHS website.^32^ For both children and adults, a diagnosis of ADHD can only be made by mental health specialists in secondary care (or through seeking a private opinion with a psychiatrist) and not by GPs. A diagnosis of depression and anxiety can be made and coded in primary care. The differential time lag in obtaining a diagnosis of ADHD versus documentation of common mental illnesses could mean that, for mid–late teens and young adults, the temporal trends in incidence observed for these two types of diagnoses might not have been affected in the same way during the pandemic. It has been reported that with the long waiting time for ADHD referrals by GPs, an increasing number of individuals have sought to obtain a diagnosis in private clinics,^33,34^ which might have contributed to the larger increase in diagnoses among those from the less deprived areas. The increase in ADHD but not in depression or anxiety disorders diagnoses may also reflect a ‘reframing’ of self-perceived mental health difficulties, which may now be more commonly viewed through the lens of neurodiversity owing to the rise in ADHD awareness and a resultant greater propensity to seek help among affected individuals and their family members.^30^

### Implications for research and practice

Early identification and timely treatment of mental health difficulties and neurodevelopmental conditions are crucial. Discussions with the PPIE group indicated that the long waiting list in the NHS was a major deterrent in accessing services, which may partly explain the fall in diagnostic and prescribing rates observed. Similar perceived barriers to accessing care affecting help-seeking decisions have also been reported by other parents and young people.^26^ The study also found a larger reduction in the use of ‘inclusive’ compared with ‘stringent’ depression codes. Future research can investigate whether this was because of the symptoms presented to health services being more severe than before the pandemic and how barriers to accessing services as a result of COVID-19 restrictions might have contributed to this trend.

PPIE members also expressed concerns that remote GP consultations, time-limited appointments, and not being able to see the same GP consistently had eroded the doctor–patient relationship and made communication more difficult. The PPIE group recommends that practices consider offering choices of consultation methods, including the option of having a parent, carer, or friend present during the appointment. The authors of this study also recommend that practices offer proactive support beyond the initial consultation and ensure doctor–patient continuity.

## Data

Electronic health records are, by definition, considered sensitive data in the UK by the Data Protection Act and cannot be shared via public deposition because of information governance restriction in place to protect patient confidentiality. Access to CPRD data is subject to protocol approval via CPRD’s Research Data Governance Process. For more information, see https://cprd.com/data-access.
